# Optimizing Noninvasive Vagus Nerve Stimulation for Systemic Lupus Erythematosus: Protocol for a Multicenter Randomized Controlled Trial

**DOI:** 10.2196/48387

**Published:** 2023-10-13

**Authors:** Ivan Contreras, Judith Navarro-Otano, Ignasi Rodríguez-Pintó, Amparo Güemes, Eduarda Alves, Roberto Rios-Garcés, Gerard Espinosa, Aida Alejaldre, Aleix Beneyto, Charrise Mary Ramkissoon, Josep Vehi, Ricard Cervera

**Affiliations:** 1 Modeling, Identification and Control Engineering (MICELab) Institut d’Informatica i Applicacions Universitat de Girona Girona Spain; 2 Professor Serra Húnter Universitat de Girona Girona Spain; 3 Neurology Service Hospital Clinic Barcelona Spain; 4 Autoimmune Diseases Unit Internal Medicine Department Hospital Universitari Mútua de Terrassa Terrassa Spain; 5 Electrical Engineering Division Department of Engineering University of Cambridge Cambridge United Kingdom; 6 Department of Autoimmune Diseases Hospital Clínic Barcelona Spain

**Keywords:** vagus nerve stimulation, autonomic nervous system, computational models, systemic lupus erythematosus, vagus, vagal, nerve stimulation, noninvasive, RCT, randomized, lupus, inflammation, autoimmune, chronic, nerve, nerve damage, vagus nerve

## Abstract

**Background:**

Systemic lupus erythematosus is a chronic, multisystem, inflammatory disease of autoimmune etiology occurring predominantly in women. A major hurdle to the diagnosis, treatment, and therapeutic advancement of this disease is its heterogeneous nature, which presents as a wide range of symptoms such as fatigue, fever, musculoskeletal involvement, neuropsychiatric disorders, and cardiovascular involvement with varying severity. The current therapeutic approach to this disease includes the administration of immunomodulatory drugs that may produce unfavorable secondary effects.

**Objective:**

This study explores the known relationship between the autonomic nervous system and inflammatory pathways to improve patient outcomes by treating autonomic nervous system dysregulation in patients via noninvasive vagus nerve stimulation. In this study, data including biomarkers, physiological signals, patient outcomes, and patient quality of life are being collected and analyzed. After completion of the clinical trial, a computer model will be developed to identify the biomarkers and physiological signals related to lupus activity in order to understand how they change with different noninvasive vagus nerve stimulation frequency parameters. Finally, we propose building a decision support system with integrated noninvasive wearable technologies for continuous cardiovascular and peripheral physiological sensing for adaptive, patient-specific optimization of the noninvasive vagus nerve stimulation frequency parameters in real time.

**Methods:**

The protocol was designed to evaluate the efficacy and safety of transauricular vagus nerve stimulation in patients with systemic lupus erythematosus. This multicenter, national, randomized, double-blind, parallel-group, placebo-controlled study will recruit a minimum of 18 patients diagnosed with this disease. Evaluation and treatment of patients will be conducted in an outpatient clinic and will include 12 visits. Visit 1 consists of a screening session. Subsequent visits up to visit 6 involve mixing treatment and evaluation sessions. Finally, the remaining visits correspond with early and late posttreatment follow-ups.

**Results:**

On November 2022, data collection was initiated. Of the 10 participants scheduled for their initial appointment, 8 met the inclusion criteria, and 6 successfully completed the entire protocol. Patient enrollment and data collection are currently underway and are expected to be completed in December 2023.

**Conclusions:**

The results of this study will advance patient-tailored vagus nerve stimulation therapies, providing an adjunctive treatment solution for systemic lupus erythematosus that will foster adoption of technology and, thus, expand the population with systemic lupus erythematosus who can benefit from improved autonomic dysregulation, translating into reduced costs and better quality of life.

**Trial Registration:**

ClinicalTrials.gov NCT05704153; https://clinicaltrials.gov/study/NCT05704153

**International Registered Report Identifier (IRRID):**

DERR1-10.2196/48387

## Introduction

### Background

The origin of autoimmune disease is a disruption of immune tolerance of self-antigens thought to occur in genetically susceptible individuals after exposure to environmental triggers. Possible environmental factors that predispose to autoimmunity include exposure to infectious agents, ultraviolet light, dietary components, chemicals, xenobiotics, toxins, and stress, as well as reduced exposure to factors that protect from the development of autoimmune diseases. Autoimmune diseases can be systemic or can affect specific organs or body systems including the endocrine system, gastrointestinal system, liver, neurological system, and musculoskeletal system and connective tissue [1].

Systemic lupus erythematosus (SLE) is a chronic systemic autoimmune disorder that commonly affects the skin, joints, kidneys, and nervous system. Abnormal activation and processing of cell death signals by the immune system trigger the release of nuclear debris from apoptotic and necrotic cells and stimulate the production of antinuclear antibodies (for example, anti-Ro and anti-DNA), with subsequent inflammation, organ damage, and pathology [2].

Many patients with SLE experience flares of disease activity interspersed with quiescent periods. The current understanding of the factors driving the different phenotypes and its pathogenesis in SLE is limited and directly affects treatment. The current therapeutic approach to SLE most commonly includes the administration of antimalarials, immunosuppressants, and biological agents. These treatments have allowed great progress to be made, and, currently, SLE-related mortality is 10% within 10 years, compared with the 1960s, when the mortality was 50% within 3 years, according to an analysis of a multisite, international SLE cohort [3]. However, these current medication options for SLE produce secondary effects, such as ocular toxicity, renal damage, risks of infections, hematological toxicities, gastrointestinal events, ovarian toxicities, and in particular, comorbidities associated with the long-term adverse effects of glucocorticoids in the musculoskeletal, cardiovascular, peripheral vascular, ocular, and metabolic domains [4].

A major hurdle to the treatment of autoimmune diseases is their heterogeneous nature. SLE is referred to as “the great imitator” because patients can experience a wide range of symptoms with varying severity [5] that appear to belong to other diseases. The heterogeneity of SLE presents challenges not only with diagnosis but also with treatment and therapeutic advancements. Broadly, manifestations of SLE include fatigue, fever, weight changes, lupus nephritis, and complications involving the following systems: musculoskeletal, gastrointestinal, pulmonary, cardiovascular, cutaneous, neuropsychiatric, hematological, and ocular.

The autonomic nervous system (ANS) is mainly composed of 2 primary branches: the sympathetic nervous system (SNS) and parasympathetic nervous system (PNS). The ANS exhibits complex central control and plays a critical role in mediating interactions between the nervous and immune systems and coordinates the interplay among cells, tissues, and organs throughout the body to maintain homeostasis via its widespread innervation of the glands, smooth muscles, and heart [6].

In many inflammatory diseases, dysautonomia manifests as an imbalance in the activity and reactivity of the SNS and PNS [7]. Increased sympathetic nerve outflow has been shown to activate pro-inflammatory cytokines and produce reactive oxygen intermediates, processes strongly implicated in the pathogenesis of SLE [2]. SLE was found to have SNS predominance or PNS dysregulation owing to decreased heart rate variability in patients with SLE. The prevalence of autonomic dysfunction ranges widely from 6% to 93% in patients with SLE [8]. Autonomic imbalance is related to an increased risk of developing cardiovascular disease—a major cause of morbidity and mortality in patients with SLE. Therefore, therapeutic strategies aimed at rebalancing the ANS may provide improved outcomes in patients with SLE.

### Prior Work

Vagus nerve stimulation (VNS) stimulates the tenth cranial nerve, the longest nerve of the organism that links the central nervous system and body by innervating major organs, such as the heart, lungs, and gastrointestinal tract. The vagus nerve is a major component of the PNS that enables bidirectional communication between the brain and different organs of the body by transmitting and exchanging sensory and motor information. The vagus nerve has been shown to play a crucial role in regulating inflammation [9] by activating the cholinergic anti-inflammatory pathway, which reduces pro-inflammatory cytokine production, inhibits pro-inflammatory activity of immune cells, and promotes the release of anti-inflammatory molecules [10]. This process is mediated by the release of acetylcholine, which binds to alpha-7 nicotinic acetylcholine receptors on immune cells, such as macrophages and T-cells, and inhibits their pro-inflammatory activity [9]. This vagus nerve–mediated pathway has been shown to have potential therapeutic applications for a range of inflammatory disorders, including rheumatoid arthritis, inflammatory bowel disease, and sepsis [11-13].

Although invasive VNS has shown positive results for the treatment of multiple diseases [11-14], VNS device implantation is associated with multiple complications, such as hoarseness, bradycardia, coughing, shortness of breath, tingling, syncope, paresthesia, muscle pain, asystole, delayed arrhythmias, sleep apnea, and surgical trauma [15-17]. To avoid implant‐related complications, noninvasive VNS (nVNS) devices that stimulate the vagus nerve through transcutaneous stimulation were developed. nVNS has shown positive therapeutic results with no significant adverse events reported for a large collection of diseases [18-28].

Varying the waveform parameters of nVNS can dramatically change the therapeutic outcome. High-frequency nVNS has been shown to stimulate different vagal fibers than those stimulated by low‐frequency nVNS, resulting in different therapeutic effects [26,28-32]. The pulse width may have a different effect on patients; a short pulse width may produce significantly less overall activation in the human brain than long pulse widths. The stimulation intensity also plays an important role and can be delivered at the subsensory, sensory, or maximal tolerance levels. Other nVNS parameters, such as the output current and signal ON and OFF times, have unknown therapeutic effects. In addition to variations in the therapeutic response, the physiological response to a given nVNS configuration can suffer from significant inter and intrapatient variability. As a result, the optimal nVNS parameters for patients with SLE are unknown. To optimize the nVNS parameters for SLE treatment, computational models can be used to study complex physiological interactions of the vagus nerve and to predict therapeutic outcomes to different nVNS configurations. Computational models have been developed for invasive VNS [33-38] and nVNS [39-41]. However, these systems have yet to be put into the context of SLE [10].

### Hypotheses and Aims

The discrepancy that exists in the use of nVNS parameters requires investigation to enable the appropriate administration of nVNS to patients with autoimmune diseases, such as SLE, for the improvement of ANS dysregulation. Therefore, the hypotheses of this study are as follows:

1. ANS dysregulation can be improved through the application of personalized nVNS.
2. At postintervention, the treatment groups (active and sham nVNS) will have different outcomes and scores.
3. Improved ANS function in patients with SLE can lead to better patient outcomes and increased quality of life.

Based on this premise, the aims of this clinical trial are as follows:

1. Collect data related to biomarkers, physiological signals, patient outcomes, and patient quality of life.
2. From the collected data, propose a computational model that can (1) identify the biomarkers and physiological signals related to SLE activity, (2) understand how the identified biomarkers and physiological signals change with different nVNS frequency parameters, and (3) include a decision support system with integrated noninvasive wearable technologies for continuous cardiovascular and peripheral physiological sensing for adaptive, patient-specific optimization of the nVNS frequency parameters in real time.
3. Validate the findings of Aranow et al [28], who found that the use of nVNS reduced pain and fatigue in patients with SLE.

## Methods

### Participants and Recruitment

Patients are recruited from the autoimmune disease outpatient clinics of the Hospital Clínic de Barcelona and Hospital Universitari Mútua Terrassa for a multicenter, national, randomized, double-blind, parallel-group, placebo-controlled, outpatient study. To compare our results with those of Aranow et al [28], a minimum of 18 adult patients (6 per study arm) is required. There is growing evidence that sex affects the pathophysiology, incidence, prevalence, development, and response to therapy in many diseases. In SLE, 90% of all diagnoses are in women [42], who are most commonly affected by clinical flares between the ages of 15 years and 44 years, or during childbearing years. Due to this disproportionality between sexes, this study aims to have a women:men ratio of 9:1, in keeping with SLE prevalence between sexes. The inclusion and exclusion criteria are summarized in Textbox 1.

**Inclusion and exclusion criteria.**

**Inclusion criteria**

1. Age ≥18 years
2. Diagnosis of systemic lupus erythematosus (SLE; defined by the American College of Rheumatology or Systemic Lupus International Collaborating Clinics [SLICC] criteria)
3. Musculoskeletal pain ≥4 on a nonanchored, 10-cm visual analog scale (VAS)
4. British Isles Lupus Assessment Group (BILAG) C on the musculoskeletal domain of the BILAG 2004
5. If on corticosteroids, stable dose of ≤10 mg/day (prednisone or equivalent) for at least 28 days before baseline
6. If on background immunosuppressive treatment, stable dose for at least 28 days before baseline
7. Able and willing to give written informed consent and comply with the requirements of the study protocol

**Exclusion criteria**

1. Treatment with rituximab within 1 year of baseline
2. Treatment with cyclophosphamide within 2 months of baseline
3. Expectation to increase steroid or immunosuppressive treatment
4. Antiphospholipid syndrome
5. Fibromyalgia (as defined by a score >13 on the fibromyalgia symptom scale)
6. Chronic fatigue syndrome
7. Treatment with an anticholinergic or sympathomimetic medication, including over-the-counter medications
8. Implantable electronic devices, such as pacemakers, defibrillators, hearing aids, cochlear implants, or deep brain stimulators
9. Joint replacement within 60 days prior to study enrollment or planned within the course of the study
10. Any planned surgical procedure requiring general anesthesia within the course of the study
11. Intra-articular cortisone injections within 28 days of the start of study
12. Chronic inflammatory disorders apart from SLE affecting the joints
13. Investigational drug or treatment during the 28 days or 7 half-lives of the investigational drug prior to the start of study drug dosing (day 0), whichever is the greater length of time
14. Active infection including hepatitis B, hepatitis C, or HIV at baseline due to high prevalence of neuropathy
15. Any condition that, in the opinion of the investigator, would jeopardize the participant’s safety following exposure to a study intervention
16. Pregnancy or lactation
17. Hemoglobin <9.0 gm/dL (according to the most recent complete blood count)
18. Comorbid disease that may require administration of corticosteroid
19. Inability to comply with study and follow-up procedures
20. Known cardiac arrhythmia, severe cardiac disease, or neurodegenerative disease
21. Peripheral or autonomic nervous system involvement, including SLE-related neuropathies, toxic polyneuropathies, metabolic neuropathies (including diabetes)
22. Previous experience with vagus nerve stimulation devices

### Screening

The screening period is set at 4 weeks and begins when the patient signs the informed consent form, which includes a full description of the study and procedures involved, patients’ rights and responsibilities, and alternative treatments that are available if the patient does not decide to participate in the study. During the screening period, the investigators must confirm that the patient meets all inclusion criteria and no exclusion criteria for the study (see the Participants and Recruitment section). Eligible patients are randomized at week 0.

Patients who are registered for this trial may have one or more active symptoms of lupus but the investigators believe that their condition is stable enough to participate. This trial design permits patients to continue their regular immune suppressive therapies at stable doses, but they are not allowed to receive additional corticosteroids during the short duration of the protocol, even if they receive a sham intervention.

### Intervention

At the second visit (week 0, baseline), after all assessments are completed and baseline laboratory samples are obtained according to the schedule of activities (see the Follow-Up section), patients are randomly assigned (1:1:1) to active (1 Hz or 30 Hz) or sham (placebo) nVNS. The rationale underlying the choice of 1 Hz and 30 Hz (minimum and maximum values of the nVNS treatment device) is based on the discrepancies found in the literature regarding the choice of a frequency value, as well as previous findings indicating that high and low frequencies of nVNS treatment stimulate different vagal fibers. The effect of VNS in epilepsy and depression is thought to be mediated through the activation of vagal afferent fibers via high-frequency (20-30 Hz) stimulation, whereas the activation of the cholinergic anti-inflammatory pathway has been found to be mediated through vagal efferent fibers involving low‐frequency (1-10 Hz) stimulation [29]. A study by Borovikova et al [30] found that VNS at 1 Hz frequency for 20 minutes was effective for the preferential recruitment of efferent parasympathetic fibers. Other reports have also shown that low-frequency (5 Hz) VNS is able to activate vagal efferent fibers [31]. In various studies of nVNS in inflammatory diseases, the amplitude was generally set individually to a constant tingling, nonpainful sensation. In these same studies, interestingly, 25 Hz [19,21,24,26] was the most frequently studied frequency, while other frequencies used were 100 Hz [23], 30 Hz [22], 5 kHz [32], and 1 Hz [12,25]. Only 2 studies have explored the low-frequency range to stimulate the vagal efferent fibers and thus, the cholinergic anti-inflammatory pathway. Straube et al [26] compared 25 Hz with 1 Hz in the treatment of chronic migraines and found a significantly larger reduction in headache days in the 1 Hz group.

Patients are blinded to the treatment allocation and are requested to refrain from discussing the details of their treatment with other patients, physicians, and clinic staff. The clinical coordinators are also blinded to the treatment allocation, and the responsible engineer instructs the patients on the proper use of the device. The study personnel have been designated to address the patient's questions and concerns as well as to record any side effects related to the use of the device. 

Active or sham nVNS is administered for 5 minutes during 5 consecutive days in a similar schedule to the treatment followed by Aranow et al [28] (5 minutes during 4 consecutive days). Clinical assessments and laboratory samples are obtained at scheduled visits according to the schedule of activities. During the treatment period, in addition to randomized treatment, patients also continue their usual medication regimen for SLE, including background standard-of-care therapy (see the Participants and Recruitment section for more details).

nVNS is administered using a Parasym device (Parasym Ltd). The Parasym device is compliant with the relevant standards contained in the Medical Device Directive 93/42/EEC. The Parasym Device is CE marked and available for purchase by individuals of the European Union.

According to the available literature, adverse effects are scarce. Although a low incidence of skin irritation has been reported from using the Parasym device, no serious adverse events have been reported in the literature. In a systematic review, Redgrave et al [43] analyzed data from 1322 participants (across 51 studies) who received nVNS. This review found that only 2.6% of participants discontinued use due to side effects, and the most common side effect reported was skin irritation at the stimulation site that typically ceased shortly after use. The low rate of cardiac arrhythmias (0.3%) is noteworthy because cardiac arrhythmias are recognized as early and late complications of invasive VNS.

### Follow-Up

Patients who complete the treatment period of the study will then attend weekly follow-up evaluations for 1 month. Subsequently, if the values of the measured biomarkers and scales of activity do not return to baseline levels (*P*<.05), additional follow-up evaluations will be performed at months 2 and 3. Patients who discontinue study treatment early will have the same posttreatment follow-up visits, unless the patient withdraws informed consent.

### Primary and Secondary Outcomes

The primary outcome of this study will be the evaluation of the causal relationship among the waveform frequency of nVNS, physiological signals, and therapeutic effect of the treatment. Therefore, we will analyze clinical and physiological changes in patients with SLE after nVNS using different waveform frequency parameters between the exploratory study at the first visit (baseline) and 30 days after the first nVNS treatment session. The secondary outcome of this study will be a comparison between our results and those of Aranow et al [28]. Both outcomes fall under the overall objective of the Modelling and Control of Non-invasive Vagus Nerve Stimulation for Autoimmune Diseases (VaNeSA) project, aimed at developing a VNS platform with an integrated VNS decision support system, including an nVNS device and portable physiological sensors that will optimize nVNS waveform parameters to maximize the therapeutic effect and minimize unwanted side effects.

The therapeutic effect and side effects will be measured using clinical, neurophysiological, and analytical tests divided into 3 sections: serum biomarkers (Textbox 2), clinical activity indices (Textbox 3), and ANS assessment (Textbox 4).

Furthermore, the addition of continuous cardiovascular and peripheral physiological sensing offers a convenient tool for this investigation owing to the intimate relationship of the vagus nerve with the heart and peripheral physiology and the ubiquity of noninvasive wearable technologies. This will allow the capture of real-time information regarding the state of the patient and their response to nVNS, which will facilitate real-time predictions of physiological responses to nVNS. Physiological signals are evaluated using a Fitbit Charge 5 activity band (Textbox 5).

**Serum biomarkers.**

1. Complete blood count (CBC)
2. Erythrocyte sedimentation rate (ESR)
3. C-reactive protein (CRP)
4. Complement C3
5. Complement C4
6. Anti-double stranded DNA (anti-dsDNA)
7. Tumor necrosis factor (TNF)
8. High mobility group box 1 (HMGB1)
9. Interleukin (IL)-6, IL-10, and IL-1B
10.  Intravesical interferon alpha (IFNα)

**Disease activity indices and outcome measures.**

1. Systemic Lupus Erythematosus Disease Activity Index (SLEDAI), British Isles Lupus Assessment Group 2004 (BILAG 2004) index
2. Physician’s Global Assessment (PGA) and Patient’s Global Assessment (PtGA)
3. 28-joint count
4. Cutaneous Lupus Erythematosus Disease Area and Severity Index (CLASI)
5. Functional Assessment of Chronic Illness Therapy – Fatigue (FACIT-F) and Fatigue Severity Scale (FSS)
6. Visual analog scale for pain (VAS Pain) and 11-point numeric rating scale
7. EQ-5D-5L, Lupus Quality of Life (LupusQoL), and Lupus Patient-Reported Outcome (LupusPRO) questionnaires

**Autonomic nervous system evaluation.**

1. Cardiovagal evaluation: continuous electrocardiogram heart rate changes during deep breathing, Valsalva maneuver, and postural changes
2. Sympathetic evaluation: beat-to-beat blood pressure changes to Valsalva maneuver and postural changes
3. Heart rate variability (HRV): continuous electrocardiogram recorded at rest for 5 minutes for HRV analysis
4. Composite autonomic symptom scale 31 (COMPASS-31)

**Physiological signals measured with a Fitbit Charge 5.**

1. Heart rate: heart rate and heart rate variability value every 5 minutes
2. Physical activity: steps, distance, and elevation every 5 minutes
3. Sleep: daily sleep duration and sleep quality

### Schedule of Activities

In total, between 10 and 12 visits are scheduled. The activities of each of these visits are detailed in Table 1. In the first visit (V1), the screening session, each patient is provided with a Fitbit Charge 5 activity band and assessed for 90 minutes. The next treatment visits (V2-V6) will last between 15 minutes and 30 minutes. We will then conduct 2 telephone follow-ups (V8 and V9) of up to 15 minutes each. Subsequently, early (V7) and late (V10) posttreatment follow-ups will be conducted for 30 minutes. Finally, if the measured biomarker values and activity scales do not return to baseline levels (*P*<.05), a very late posttreatment follow-up of 1 or 2 visits (V11 and V12), each lasting 30 minutes, will be performed.

**Table 1:** Activities of the study during the scheduled visits.

Details		Visit number											
		V1	V2	V3	V4	V5	V6	V7	V8	V9	V10	V11^a^	V12^a^
Study day		–28	1	2	3	4	5	8	15	22	29	56	84
**Activities**													
	Informed consent	✓											
	Wearable	✓											
	Demographics	✓											
	Medical history	✓											
	Physical examination	✓	✓	✓	✓	✓	✓	✓			✓	✓	✓
	Substance use^b^	✓	✓					✓			✓	✓	✓
	Nervous system evaluation	✓						✓			✓	✓	✓
	Concomitant medications	✓	✓	✓	✓	✓	✓	✓	✓	✓	✓	✓	✓
	Review inclusion and exclusion criteria	✓	✓										
	Adverse events		✓	✓	✓	✓	✓	✓	✓	✓	✓	✓	✓
	Randomization		✓										
	Investigational treatment administration		✓	✓	✓	✓	✓						
	SLEDAI-2K^c^	✓	✓	✓	✓	✓	✓	✓			✓	✓	✓
	BILAG-2004^d^		✓									✓	✓
	PGA^e^	✓	✓	✓	✓	✓	✓	✓	✓	✓	✓	✓	✓
	PtGA^f^	✓	✓	✓	✓	✓	✓	✓	✓	✓	✓	✓	✓
	CLASI^g^	✓	✓	✓	✓	✓	✓	✓			✓	✓	✓
	Tender/swollen joint count	✓	✓	✓	✓	✓	✓	✓			✓	✓	✓
	FACIT-F^h^	✓	✓	✓	✓	✓	✓	✓	✓	✓	✓	✓	✓
	FSS^i^	✓	✓	✓	✓	✓	✓	✓	✓	✓	✓	✓	✓
	VAS^j^ Pain	✓	✓	✓	✓	✓	✓	✓	✓	✓	✓	✓	✓
	11-point NRS^k^ scale	✓	✓	✓	✓	✓	✓	✓	✓	✓	✓	✓	✓
	EQ-5D-5L		✓	✓	✓	✓	✓	✓	✓	✓	✓	✓	✓
	LupusQoL^l^		✓	✓	✓	✓	✓	✓	✓	✓	✓	✓	✓
	LupusPRO^m^		✓	✓	✓	✓	✓	✓	✓	✓	✓	✓	✓
	COMPASS-31^n^	✓						✓			✓		
	CBC^o^	✓						✓			✓	✓	✓
	ESR^p^ and CRP^q^	✓						✓			✓	✓	✓
	C3^r^, C4^s^, anti-dsDNA^t^	✓						✓			✓	✓	✓
	TNF^u^, HMGB1^v^, IL^w^-6, IL-1B, IL-10	✓						✓			✓	✓	✓
	IFNα^x^	✓						✓			✓	✓	✓

^a^Only if the values of the measured biomarkers and scales of activity do not return to baseline levels (*P*<.05).

^b^Caffeine, alcohol, or tobacco.

^c^SLEDAI-2K: Systemic Lupus Erythematosus Disease Activity Index.

^d^BILAG 2004: British Isles Lupus Assessment Group 2004.

^e^PGA: Physician’s Global Assessment.

^f^PtGA: Patient’s Global Assessment.

^g^CLASI: Cutaneous Lupus Erythematosus Disease Area and Severity Index.

^h^FACIT-F: Functional Assessment of Chronic Illness Therapy – Fatigue.

^i^FSS: Fatigue Severity Scale

^j^VAS: visual analog scale.

^k^NRS: numeric rating scale.

^l^LupusQoL: Lupus Quality of Life.

^m^LupusPRO: Lupus Patient-Reported Outcome.

^n^COMPASS 31: Composite autonomic symptom scale 31.

^o^CBC: complete blood count.

^p^ESR: erythrocyte sedimentation rate.

^q^CRP: C-reactive protein.

^r^C3: complement C3.

^s^C4: complement C4.

^t^anti-dsDNA: anti-double stranded DNA.

^u^TNF: tumor necrosis factor.

^v^HMGB1: high mobility group box 1.

^w^IL: interleukin.

^x^IFNα: interferon alpha.

### Statistical Analysis

The Wilcoxon rank sum test will be used to compare the change in endpoints from baseline to day 7 and from baseline to day 21 in participants receiving active and sham nVNS. ﻿Spearman rank-order correlation will be used to assess the potential associations between endpoints. False discovery rate control will be used to mitigate the problem of a false positive rate error. Statistical analysis will be performed using the SciPy Python-based library.

### Ethics Approval

This study is being conducted in compliance with the Declaration of Helsinki (Fortaleza, Brazil, October 2013). The study is being carried out in accordance with the protocol and with the relevant legal requirements, Law 14/2007 of July 3, on Biomedical Research. The study was approved by the Clinical Research Ethics Committee of the Hospital Clínic of Barcelona (protocol identifier: HB/2021/1286; approved March 14, 2022). Informed consent is obtained from all participants invited to participate in the study prior to the baseline visit. Data will be processed in accordance with ethical principles that guarantee the anonymity of the participants.

## Results

The study is supported by the Spanish Ministry of Science and Innovation through grant PID2020-117171RA-I00 funded by MCIN/AEI/10.13039/501100011033 in July 2021. The data collection began on November 19, 2022. The clinical study staff have been engaged in activities including enrollment and data collection. As of March 1, 2023, a total of 10 participants had been admitted for their first appointment, 8 had met the inclusion criteria, and 6 had completed the treatment. Recruitment and data analysis are expected to be completed in December 2023.

## Discussion

### Overview

This study belongs to a collection of studies pertaining to the VaNeSA project. The overall goal of the VaNeSA project is to develop an nVNS platform with nVNS, an integrated nVNS decision support system, and physiological wearable sensors that will optimize nVNS waveform parameters to maximize the therapeutic nVNS effect while minimizing unwanted side effects.

This clinical trial is the first conducted in the VaNeSA project that will analyze the physiological signals, biomarkers, and clinical outcomes to determine the most effective measures to monitor, optimize, and personalize nVNS in patients with SLE. The ultimate aim of this study is to confirm that the application of personalized nVNS will improve ANS dysregulation, a possible driver of autoimmune disease, leading to better patient outcomes and quality of life. After this study, computational models to describe and predict the impact of nVNS on the ANS and clinical outcomes for patients with SLE will be created, implemented, and validated. The goal of developing computational models is the objective not only of this study but also of the framework project (VaNeSA project) in which there will be more clinical experimentation with other related diseases and other sets of nVNS parameters. The results obtained in this clinical trial will serve to determine the most appropriate approach for obtaining these models, but the idea is to condition the physiological responses of the treatment to the therapy administered so that we can develop a first prototype to induce the application of specific stimulation parameters to each patient. Although the creation of computational models depends on the overall quality of the final results, the initial plan is to use modelling algorithms using artificial intelligence techniques. We plan to implement a decision tree–based methodology since it seems to be one of the best alternatives, as their conditional control statements decompose complex data into more manageable parts. The validated computer models, a decision support system, and wearable physiological sensors will be integrated into an nVNS platform allowing for the optimization and personalization of nVNS therapy in real time. Finally, the nVNS platform will be tested and validated.

### Limitations and Strengths

This randomized, double-blinded, placebo-controlled clinical trial is one of the first studies delving into the impact of nVNS on clinical outcomes for patients with SLE. The design is a parallel study used to ensure optimal blinding and adjust to the time constraints of the study. However, this study design is weaker than that of a crossover design. The nVNS sessions are being performed by the hospital staff, potentially limiting the willingness of patients to enroll in the study due to the frequency of required hospital visits but ensuring correct treatment delivery and safety (sham treatment, stimulation intensity, localization, and duration). Evidence from this trial will have a limited effect on the knowledge of the optimization of the nVNS parameters because only the frequency domain is evaluated while the remaining characteristic parameters of the nVNS will remain unknown. The small sample size may also pose a limitation in terms of sample representation and statistical power. Finally, in comparison with prior research, our study involves a comprehensive number of biomarkers and activity indices, including ANS and PNS evaluations, that will provide extensive baseline characterization and explore dose-response characteristics of nVNS, allowing nVNS to be contextualized for clinical use.

### Conclusions

This study addresses the potential use of nVNS as an adjunctive treatment for SLE. The goal of the study is to provide information on the use of nVNS in SLE and move toward a patient-tailored therapeutic solution that can encourage the adoption of VNS technologies and thus expand the SLE population that can benefit from improved autonomic dysregulation, resulting in reduced costs and improved patient outcomes and quality of life.

## Abbreviations

nVNS: noninvasive vagus nerve stimulation
PNS: parasympathetic nervous system
SLE: systemic lupus erythematosus
SNS: sympathetic nervous system
VaNeSA: Modelling and Control of Non-invasive Vagus Nerve Stimulation for Autoimmune Diseases
VNS: vagus nerve stimulation

## Data Availability

Deidentified participant data will be shared upon reasonable request after completion of the trial and publication of the study results. Data will be available for use if the request is approved by the appropriate institutional review board and the Trial Steering Committee and a signed data access agreement is in place.
